# Neural-network-backed evolutionary search for SrTiO_3_(110) surface reconstructions†

**DOI:** 10.1039/d2dd00072e

**Published:** 2022-08-26

**Authors:** Ralf Wanzenböck, Marco Arrigoni, Sebastian Bichelmaier, Florian Buchner, Jesús Carrete, Georg K. H. Madsen

**Affiliations:** Institute of Materials Chemistry, TU Wien 1060 Vienna Austria georg.madsen@tuwien.ac.at

## Abstract

The determination of atomic structures in surface reconstructions has typically relied on structural models derived from intuition and domain knowledge. Evolutionary algorithms have emerged as powerful tools for such structure searches. However, when density functional theory is used to evaluate the energy the computational cost of a thorough exploration of the potential energy landscape is prohibitive. Here, we drive the exploration of the rich phase diagram of TiO_*x*_ overlayer structures on SrTiO_3_(110) by combining the covariance matrix adaptation evolution strategy (CMA-ES) and a neural-network force field (NNFF) as a surrogate energy model. By training solely on SrTiO_3_(110) 4×1 overlayer structures and performing CMA-ES runs on 3×1, 4×1 and 5×1 overlayers, we verify the transferability of the NNFF. The speedup due to the surrogate model allows taking advantage of the stochastic nature of the CMA-ES to perform exhaustive sets of explorations and identify both known and new low-energy reconstructions.

## Abstract 2

The covariance matrix adaptation evolution strategy (CMA-ES) and a fully automatically differentiable, transferable neural-network force field are combined to explore TiO_*x*_ overlayer structures on SrTiO_3_(110) 3×1, 4×1 and 5×1 surfaces.

## Graphical abstract

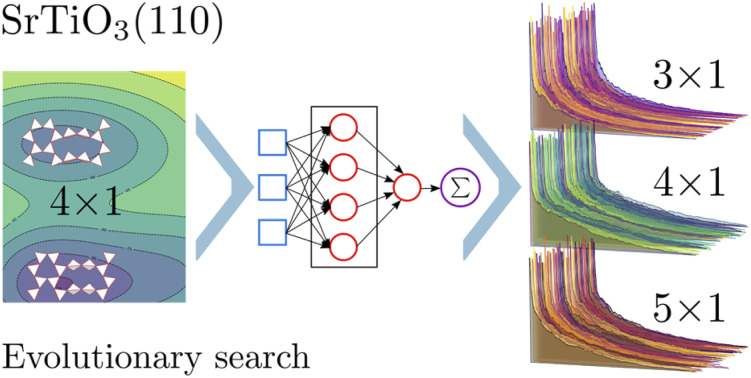

## 1 Introduction

The determination of surface structure is vital in gaining a better understanding of the properties and possible applications of materials. Traditionally, being able to identify the true atomic structure of materials from experimental findings is an acquired skill, based on experience and domain knowledge.^1–4^ Due to its semiconducting nature and possible applications in electronic devices, strontium titanate (SrTiO_3_) has increasingly been the focus of experimental and theoretical studies.^5–10^ Since many possible applications of SrTiO_3_ are realized in the form of nanoscale thin films, its surfaces are of particular interest.

Here, we are interested in exploring the phase diagram of TiO_*x*_ overlayer structures on SrTiO_3_(110) under Ti-poor conditions.^11^ The polar nature of the bulk terminated SrTiO_3_(110) surfaces is balanced by a Ti_*n*+2_O_3*n*+4_ overlayer with a *n*×1 unit cell.^2^ The *n* increases systematically with Sr chemical potential^11^ and an early model, based on simple geometric considerations and DFT calculations, proposed a systematic set of overlayer structures consisting of a ring of six Ti–O tetrahedra and an additional ring of increasing length so that a *n* = 2 unit cell would exhibit a 6–6 overlayer, a *n* = 3 unit cell a 6–8 overlayer and so on and so forth.^2^ Subsequently, DFT investigations refined the picture^3,12–14^ and showed, *e.g.*, how an 8–10 overlayer structure is stable in the 5×1 cell.^12^ However, despite the decade-long interest, the understanding of the phase diagram is still based on the investigation of specific structures derived from experience and geometric considerations.

The wide availability of first-principles computations based on density functional theory (DFT), combined with the steadily increasing computational power, have greatly improved the feasibility of global geometry optimization. Evolutionary algorithms^15,16^ combined with these first-principles calculations have proven to be powerful tools for structure searches,^17–22^ including the investigation of surface reconstructions.^23^ However, the stochastic nature of the corresponding algorithms means that the computational cost and run-time associated with a thorough exploration of the potential energy surface (PES) quickly strain available computational resources. With the growing availability of machine-learned (ML) approximations of the PES^24^ various incarnations have been successfully adapted for use with evolutionary algorithms in structure prediction^25,26^ and surface reconstructions.^27,28^ Due to the large variety of structures that can potentially be visited during an evolutionary search, the construction of a suitable ML PES approximation is very challenging. Adaptive approaches have been used where the ML approximation is updated every time a point of high uncertainty is visited.^28^ However, the estimation of uncertainty is still a relatively open question in ML.^29^ Alternatively, the ML PES should be trained on a highly diverse set of structures.^27^ In most cases though, this is not practically feasible and gathering training data from the entire PES *a priori* would also somewhat defy the objective of making an ML PES approximation.

In the present work we perform an exploration of the TiO_*x*_ overlayer structures by combining the covariance matrix adaptation evolution strategy (CMA-ES)^30^ and a fully automatically differentiable neural-network force field (NNFF).^31^ First, we discuss how an implementation of the CMA-ES is adapted to deal with surface structures. We use the initial DFT based CMA-ES run to set up a diverse set of training data and construct an NNFF. We train the initial NNFF solely on SrTiO_3_(110) 4×1 overlayer structures and perform CMA-ES runs on 3×1, 4×1 and 5×1 overlayers. We verify the transferability of the NNFF in a diagnostic approach by constructing NNFFs including data from the overlayers not part of the original training dataset and utilizing similarity in the local environments. With this we are able to investigate larger systems, while performing the majority of expensive DFT calculations on more accessible structures. Furthermore, the NNFFs make it possible to perform sets of CMA-ES runs, taking advantage of the stochastic nature of the algorithm to more fully explore the PES. We show how the procedure reproduces known structures and also finds new stable structures for all three overlayers considered.

## 2 Methods

### 2.1 Slab setup

**Fig. 1:**
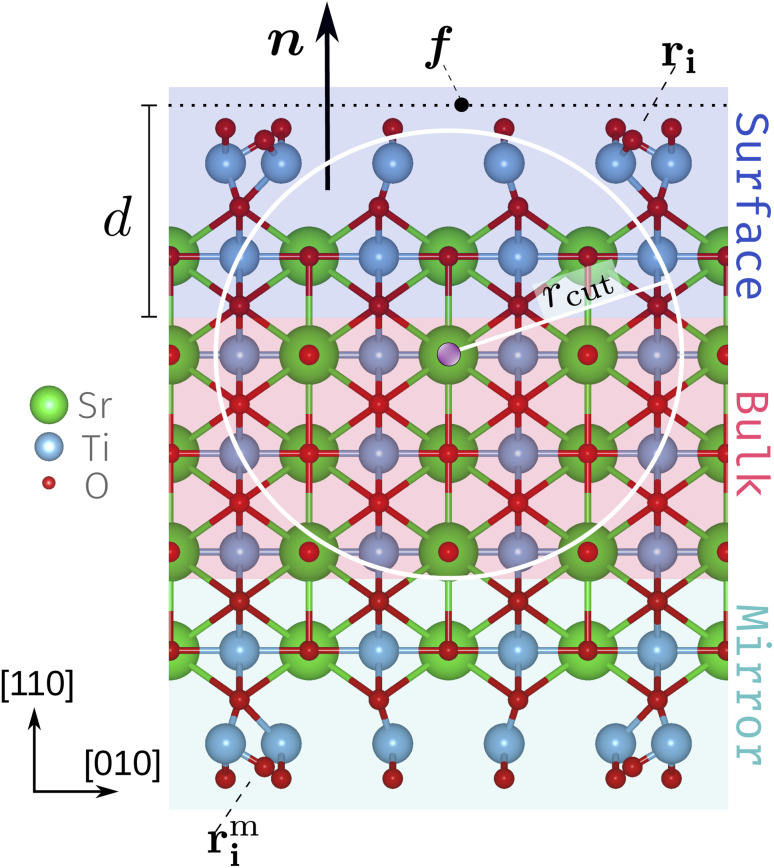
Side view of an SrTiO_3_(110) 4×1 founder slab. The schematic diagram includes the surface normal ***n***, defect focus ***f***, depth *d* and the surface atoms ***r***_*i*_ with their mirrors images ***r***^m^_i_. The white circle and radius *r*_cut_ represent a 2D slice of the local environment of the atom highlighted in purple. The fixed bulk-like anchor (red), the surface layers that can be accessed by the CMA-ES algorithm (blue) and their mirror layers (green) highlighted. The highlighting corresponds to a choice of *d* = 6 Å.

We use a slab setup starting from a bulk-terminated structure composed of five SrTiO and six O_2_ alternating layers with vacuum separating the surface atoms from the periodic recurrence of the slab along the surface normal. To both sides of the slab roughly evenly spaced Ti_2_O_5_ and TiO_2_ units were added according to the stoichiometry Ti_*n*+2_O_*n*+4_ for unit cells with *n*×1 periodicity. With this the SrTiO_3_(110) 3×1, 4×1 and 5×1 unit cells contain 105, 136 and 167 atoms each in total. For the subsequent CMA-ES runs we refer to these structures as the founder structures, Fig. 1 (see also deposited data^32^). The outermost O_2_ layer and added Ti–O units comply with the target composition Ti_*n*+2_O_3*n*+4_^2^ and are referred to as the TiO_*x*_ overlayers. The atoms in the innermost region (red background in Fig. 1) are fixed to act as an anchor and the opposite sides of the slab are symmetrical. The mirror atoms' positions ***r***^m^_i_ are obtained from the original positions ***r***^m^_i_ by a point inversion about the center of the slab. The width of the fixed bulk layer (three SrTiO and two O_2_ layers in Fig. 1) is controlled by a computational parameter (the depth *d*) discussed below.

### 2.2 CMA-ES

The CMA-ES^30,33^ draws a sample of *λ* individuals ***x***^(g)^_k_, *k* = 1, ..., *λ* at each generation *g* from a multivariate normal distribution:1
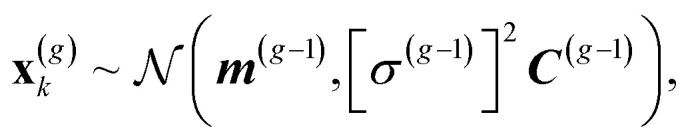
where ***m*** represents the distribution mean, *σ* the step size and ***C*** the covariance matrix. The distribution is iteratively adapted by the algorithm after each generation through the use of so-called evolution paths,^33^ which are able to take into account the evolutionary history in the parameters update.

We employ the standard hyperparameter settings,^33^ including the initialization of the covariance matrix to identity and choosing the population size *via λ* = 4 + ⌊3 log *D*⌋, where *D* are the degrees of freedom. The initial step size, which controls the width of the initial distribution, is set to *σ*^(0)^ = 0.12 Å.

We recently implemented the CMA-ES for point defect structural exploration.^26^ Here, we extend the implementation to efficiently work on surface structures. The CMA-ES directly determines the positions of only the surface atoms (overlayer and outermost bulk, highlighted in blue in Fig. 1) ***r***_*i*_; the mirror atoms ***r***^m^_i_ on the opposite side (highlighted in green) are adjusted accordingly. To specify which atoms the algorithm has access to, the surface normal ***n***, a defect focus ***f*** (on a plane perpendicular to ***n***) and a depth d[Å] need to be defined. In this work we set the defect focus such that it marks the lower edge of the surface slab along the c-axis and the surface normal as ***n*** = (0, 0, 1). With these settings constant, solely the depth *d* controls which atoms the algorithm has access to.

### 2.3 DFT

We utilize GPAW^34^ in linear-combination-of-atomic-orbitals (LCAO)^35^ mode as the energy backend for the first CMA-ES runs and for the local gradient based optimizations and, furthermore, for the generation of training data and evaluation of structures gathered from NNFF-backed CMA-ES runs. All GPAW calculations are performed employing the Perdew–Burke–Ernzerhof (PBE)^36^ functional. Simulation boxes with periodic boundary conditions perpendicular to the surface normal are used for the GPAW calculations and the *k*-point grid is set to (2, 2, 1). For reference some structures were also optimized locally using VASP^37^ with periodic boundary conditions applied along all axes. Several individuals are optimized locally *via* the FIRE^38^ algorithm, built into the atomic simulation environment (ASE).^39^

To investigate the diversity of the training data, we calculated the net atomic charges (NAC) using the Chargemol^40^ program, which implements the DDEC6 approach.^41,42^

### 2.4 NNFF

We train NNFFs following the NeuralIL methodology.^31^ The implementation is based on JAX,^43^ which offers just-in-time compilation and end-to-end automatic differentiation allowing us to train on all 3 *n*_atoms_ forces within each structure. The Cartesian coordinates are encoded using the power spectrum of element-specific atom-centered spherical Bessel descriptors^31,44,45^ and atom types are factored in *via* an embedding layer.^31,46^ The importance of encoding element specific environments for multicomponent systems was previously pointed out.^31^ In the present work, these were encoded with a resolution set by *n*_max_ = 5 within a cutoff radius of *r*_cut_ = 5.5 Å, resulting in 126 descriptors per atom. For all models we use a fully connected pyramidal architecture with hidden layers consisting of 256:128:64:32:16:16:16 nodes.

The training is performed over 350 epochs with a one-cycle learning rate schedule^47^ varying the learning rate linearly from 3×10^−4^ to 3×10^−3^ and back, and switching to 3×10^−5^ for the last 10% of each epoch, with the data being split into minibatches of eight structures each. To reduce the influence of outliers, the log-cosh loss function^48^ is employed, with a characteristic scale parameter of 0.1 eV Å^−1^, effectively clipping very large forces.

## 3 Results and discussion

We first performed three CMA-ES runs with GPAW as the fitness backend. All runs started from the same founder structure with the depth *d* being switched from 3 to 6 and to 9 Å, see Fig. 1. This allowed the CMA-ES to manipulate the positions of atoms in only the TiO_*x*_ overlayer for *d* = 3 Å, additionally one SrTiO layer and one O_2_ layer for *d* = 6 Å (as depicted in Fig. 1) and all but the central SrTiO layer for *d* = 9 Å. This corresponded to totals of 22, 42 and 62 atoms, respectively. Each run produced 500 generations of 22 individuals, totaling 33 000 structures among the three runs.

**Fig. 2:**
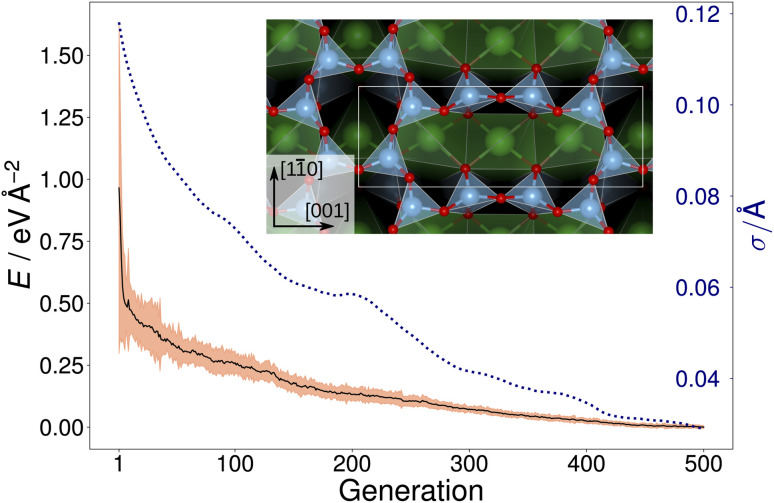
Trajectory (black solid line) of the average energy of the population over 500 generations of a GPAW-backed CMA-ES run on a SrTiO_3_(110) 4×1 structure with *d* = 6 Å. The three times the standard deviation of energies within each generation is highlighted in orange. The area highlighted in orange has a height equal to three times the standard deviation of the energies within each generation. The dotted blue line shows the CMA-ES parameter step-size *σ*. In the inset, the local optimization of the resulting overlayer consisting of rings of corner-sharing TiO_4_ tetrahedra is shown; the unit cell is indicated by a white rectangle.

Fig. 2 shows the trajectory of the average energy of the populations and the CMA-ES step size. The step size, *σ*, was automatically adjusted by the algorithm, narrowing or widening the underlying distribution [eqn. (1)] as necessary. The inset in Fig. 2 displays the overlayer reconstruction of a SrTiO_3_(110) 4×1 surface that was identified by local optimization of the lowest energy structure generated by the CMA-ES for depth *d* = 6 Å. The structure reproduces the expected overlayer with rings of corner-sharing TiO_4_ consisting of six and ten members, respectively.^2,3^ However, it does not represent the ground-state overlayer structure, as will be discussed later.

**Fig. 3:**
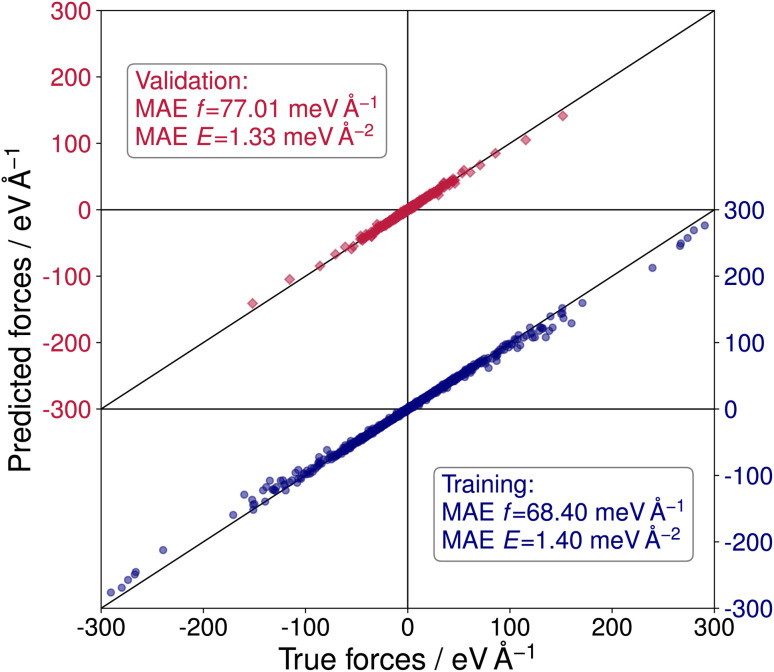
Predicted *vs.* true forces for the NeuralIL model NNFF_1_ on training (blue dots) and validation data (red squares). MAEs are given for forces and energies.

To perform sets of multiple evolutionary runs within reasonable time frames and, thereby, utilize the stochastic nature of the CMA-ES algorithm to explore more distinct local minima of the PES, a NNFF model was trained on selected SrTiO_3_(110) 4×1 structures. The training data was obtained from the three DFT-based CMA-ES trajectories described above. From these we used the individual structure with the lowest energy and a randomly chosen structure of each generation of each run. For the validation data, an individual from each generation was chosen at random. With this we arrived at 3000 configurations in the training data and 500 in the validation data. Finally, 5000 structures were chosen completely at random from the available DFT calculations as test data, only excluding previously selected data. The resulting model, labeled NNFF_1_, achieved a mean absolute error (MAE) of 1.79 meV per atom or in terms of surface energy 1.40 meV Å^−2^ for training energies and 68.40 meV Å^−1^ for training forces. The NNFF performance on training and validation data is shown in Fig. 3, which does not show any indication of overfitting. Similar MAEs were also found for the test set, labeled 4×1 in Table 1.

**Table 1:** Performance of three trained NeuralIL models on distinct test data sets (5000 4×1 structures, 1100 5×1 structures, 149 GPAW-optimized 5×1 structures), comparing the MAEs of the force components and of the energies per surface area

Test set	MAE	NNFF_1_	NNFF_2_	NNFF_3_
4×1	*f*/meV Å^−1^	77.19	84.22	79.76
	*E*/meV Å^−2^	1.35	2.07	1.61
5×1	*f*/meV Å^−1^	278.55	123.46	126.37
	*E*/meV Å^−2^	9.89	1.92	2.21
Opt	*f*/meV Å^−1^	175.40	105.35	107.66
	*E*/meV Å^−2^	8.95	7.72	6.59

**Fig. 4:**
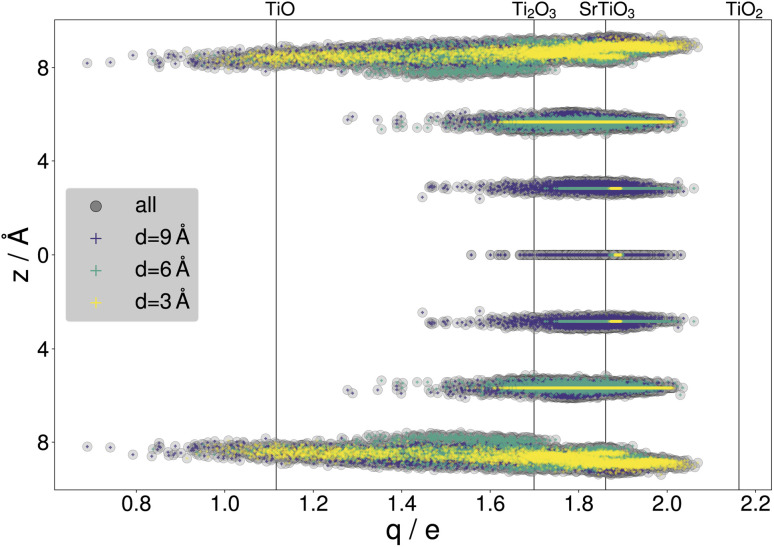
Net atomic charges of titanium atoms within the SrTiO_3_(110) 4×1 training structures gathered from all three DFT-backed searches. The Ti charges are highlighted according to the depth parameter, with results for *d* = 3 Å in yellow, *d* = 6 Å in green and *d* = 9 Å in purple. *z* = 0 Å marks the SrTiO layer in the center of the surface slabs. The vertical lines indicate charges calculated for bulk systems with titanium in different oxidation states: TiO, Ti_2_O_3_, TiO_2_ (rutile) and SrTiO_3_.

By gathering the data from CMA-ES runs, a large variety of structures were included in the data sets, with a standard deviation of 201.11 meV per atom or 150.08 meV Å^−2^ in the training energies and 2.35 eV Å^−1^ in the training force components, with maximal training forces of up to 290.53 eV Å^−1^. The diversity of the training data is illustrated with the net atomic charges^41,42^ on titanium. Fig. 4 compares the resulting charges to known titanium oxidation states. It is seen that the charges within the surface slabs cover an even larger range than the illustrated reference systems. The trained NNFF_1_ did not show any issues handling the different oxidation states.

With the goal of a broader exploration of the respective PES, a set of 50 CMA-ES evolution runs was performed on SrTiO_3_(110) 3×1, 4×1 and 5×1 surfaces utilizing NNFF_1_. We selected *d* = 6 Å thus allowing the CMA-ES to manipulate two SrTiO_3_ layers. Within a set the same founder structure was used for all runs, whereas the initial random seed of the CMA-ES was varied. For all runs the maximum number of generations was set to at least 500, with the additional stopping criterion of the standard deviation of the individuals' energies within a generation going below 50 meV per unit cell. Every generation of 4×1 and 5×1 structures consisted of 22 individuals, while this number was automatically set to 21 by the algorithm for 3×1 due to the lower number of atoms in the structure. Otherwise, all CMA-ES parameters were identical for all investigated systems. This led the algorithm to arrive at a diverse collection of individuals belonging to a number of different local minima on the PES for each system. The best individual of each CMA-ES run within a set was optimized locally using first NNFF_1_ and subsequently GPAW as the backend.

**Fig. 5:**
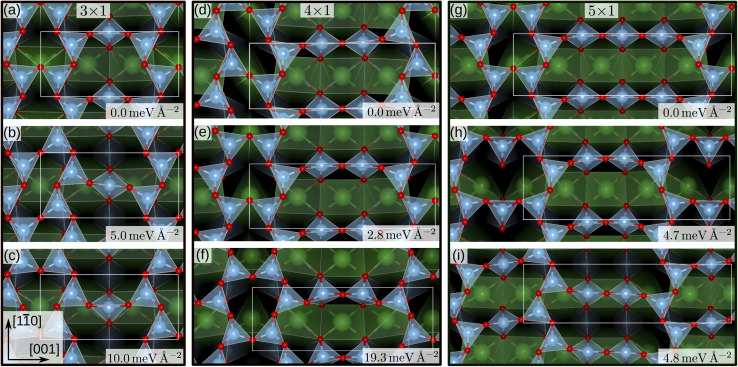
SrTiO_3_(110) 3×1, 4×1 and 5×1 reconstruction overlayers identified by performing sets of NNFF-backed CMA-ES runs and further refined by two subsequent optimizations, driven by the NNFFs and GPAW. All structures show corner-sharing TiO_4_ tetrahedra in different arrangements of six-, eight-, ten- or twelve-membered rings. For each of the system sizes the calculated energy minimum is set to zero. Structures (a), (e) and (g) reproduce overlayers in agreement with literature.^2^ The energies shown were evaluated using GPAW and offset to the computational minimum per reconstruction.

**Fig. 6:**
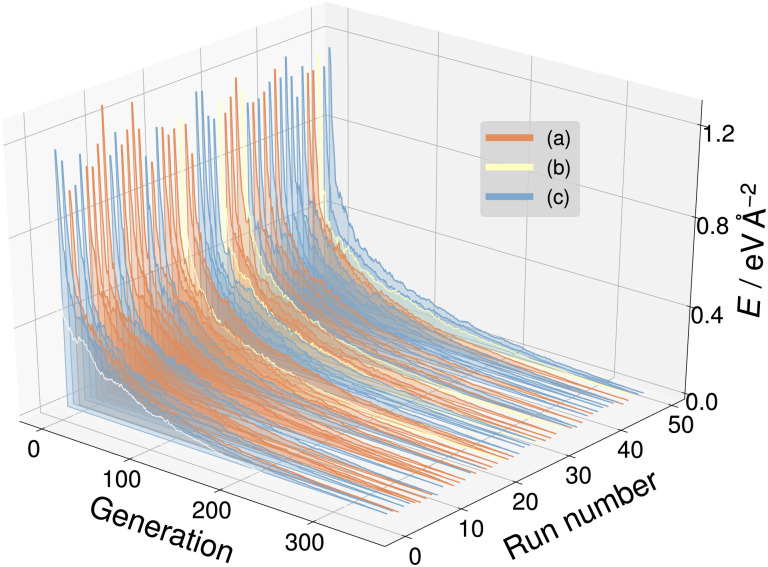
The energy trajectories of the 50 CMA-ES runs on the SrTiO_3_(110) 3×1 surface, with the calculated energy minimum set to zero. The labels (a) to (c) correspond to the overlayers shown in Fig. 5(a)–(c). Run number zero (white) is an outlier.

For the SrTiO_3_(110) 3×1 surface slab, 49 of the 50 candidate structures fell on three distinct energy levels corresponding to the overlayer reconstructions shown in Fig. 5(a)–(c). The corresponding CMA-ES energy trajectories are shown in Fig. 6, with four runs arriving at structure (b) and the rest evenly distributed between (a) and (c). All three 3×1 overlayers include six- and eight-membered rings of corner-sharing TiO_4_ tetrahedra, with the smaller ring taking triangular or rhombic shape. The TiO_*x*_ overlayers in (a) and (c) are related by a shift of half a lattice unit along the [11̄0] direction, which results in a different connectivity to the substrate. As expected from the literature,^2^ reconstruction (a) arrived at the lowest energy. Additionally, one structure for each of the three reconstructions pictured in Fig. 5(a)–(c) was further optimized using VASP. The overlayers did not change through the VASP optimization and the relative order of the energies was the same: with the minimum VASP energy of (a) set to zero, (b) arrived at 4.8 meV Å^−2^ and (c) at 5.9 meV Å^−2^.

The same strategy was applied to SrTiO_3_(110) 4×1 surfaces. Here, the GPAW-backed optimization of the 50 best individuals identified two distinct low-energy overlayers presented in Fig. 5(d) and (e), with the rest of the structures spanning an energy range of 100 meV Å^−2^. The configuration with the third-lowest energy, shown in Fig. 5(f) interestingly reproduces the result of the DFT-backed CMA-ES, Fig. 2, which underlines the need for running multiple sets of PES explorations. All three overlayers, Fig. 5(d–f), showed corner-sharing TiO_4_ tetrahedra arranged in rings of six and ten members, with the smaller ring taking the shape of a rhombus (d) or a triangle (e) and (f), respectively. Structure (e) is the expected stable structure.^2,3^ However, overlayer (d) represents the computational energy minimum and suggests an alternative organization of the overlayer, exhibiting a *p*2 symmetry in contrast to the mirror symmetry in (e) and (f). The rhombohedral structural motif of the six-membered ring is similar to that of the 3×1 candidate in Fig. 5(b). Notably, regions exibiting *p*2 symmetry can be identified in STM images published by Wang *et al.* as Fig. 1(a),^14^ near Type-II vacancies. To illustrate this observation a simulated STM image of the structure in Fig. 5(d) was generated utilizing the Tersoff–Hamann approximation.^49^ The result can be seen in Fig. 1 of the ESI together with the experimental STM image and indeed a very good agreement is observed. Configurations representing each minimum were optimized with VASP. The overlayer structures remained unchanged. The energy differences became smaller, with (d) and (e) differing by only 0.08 meV Å^−2^ and (f) lying 9.7 meV Å^−2^ higher, but the order remained the same.

Finally, we performed a set of 50 CMA-ES runs for the SrTiO_3_(110) 5×1 surfaces. The GPAW-optimized CMA-ES results identified a number of overlayers consisting of corner-sharing TiO_4_ tetrahedra arranged in rings with structures representing variations of six- and twelve-membered rings or eight- and ten-membered rings. The lowest-energy structures are shown in Fig. 5(g), a six-twelve structure proposed in literature,^2^ and in (i), an eight–ten structure with *p*2 symmetry with a 4.8 meV Å^−2^ higher energy than (g). To the best of our knowledge the *p*2 structure in (i) has not been proposed before and we have deposited the calculated STM image in Fig. 2 of the ESI†.

The predictions of ML models can be expected to have the largest uncertainty for data with a low similarity to the training data.^29^ Intuitively, the structures with the lowest similarity to the 4×1 training data would be found among the structures visited in the 5×1 searches. To investigate the reliability of the NNFF we trained a new NNFF by adding 5×1 structures to the training data. To that end, 3500 out of the 550 000 available 5×1 structures generated by the 50 NNFF_1_ CMA-ES runs were selected randomly for evaluation with GPAW. We then gathered 4000 structures as training data and 800 for validation, evenly distributed in 4×1 and 5×1 data points. The remaining 1100 new 5×1 DFT data were collected in a second test set, labeled 5×1 in Table. 1. The resulting model was labeled “NNFF_2_′′ and achieved an MAE on training forces of 83.53 meV Å^−1^ and 93.96 meV Å^−1^ for validation. The force and energy MAEs for NNFF_1_ and NNFF_2_ for the two test sets are given in Table. 1. As expected, the performance on a test set exclusively containing 5×1 structures improved significantly, with NNFF_2_ achieving an MAE of 123.5 meV Å^−1^ on the forces, in comparison to 278.6 meV Å^−1^ for NNFF_1_. The performance on the 4×1 test set deteriorates somewhat, from 77.2 meV Å^−1^ to 84.2 meV Å^−1^. This, however, is expected because of the reduced number of 4×1 structures in the training data.

**Fig. 7:**
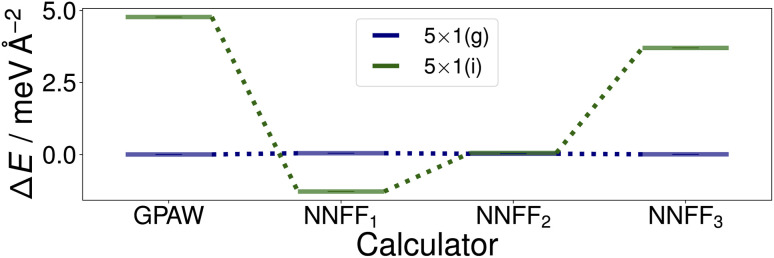
Surface energies of the (g) and (i) structures, Fig. 5, identified by the first two sets of evolution runs performed on the SrTiO_3_(110) 5×1 surface. The energies of the optimized structures GPAW structures (first column) were evaluated with the three trained models. The energies shown are offset to that of structure (g).

Utilizing NNFF_2_, we performed a second set of 50 CMA-ES runs on the 5×1 surface slab, which again resulted in the low-energy structures (g) and (i) in Fig. 5. This can be seen as a confirmation of the transferability of the NNFF_1_ model. However, it is also clear that the energy differences between the structures (g) and (i) are on the same scale as the MAE of both NNFF_1_ and NNFF_2_. When evaluating the energies of the two structures we also see that NNFF_1_ swaps their order, while with NNFF_2_ they become almost energetically degenerate, as seen in Fig. 7. So while both NNFFs are able to distinguish the two basins, it is an open question how much the energy uncertainty influences the CMA-ES exploration of the PES.

To investigate this question, a third data set was created by pooling all previously used 4×1 and 5×1 training and validation structures and augmenting the data with 350 5×1 structures representing the minima found. These 350 additional structures were randomly selected from the GPAW optimization trajectories of seven CMA-ES results, two from set one (NNFF_1_) and five from set two (NNFF_2_), representing various energy basins. The resulting data was then randomly split into 5000 training data and 1250 validation data and the model, labeled “NNFF_3_”, trained over 500 epochs with all other parameters unchanged, achieving an MAE on training forces of 76.90 meV Å^−1^ and 87.69 meV Å^−1^ for validation. By pooling the whole collection of 4×1 and 5×1 data, the model performed well on both the 4×1 and 5×1 test sets, Table. 1. We also constructed a new test set, denoted as “Opt” in Table. 1, consisting of the GPAW converged minima of all three CMA-ES sets that were performed on 5×1 structures. Not surprisingly, NNFF_3_ performed well for this test set. Importantly, NNFF_3_ was able to correctly reproduce the order of the lowest energy structures found for the 5×1 reconstruction, Fig. 7. Subsequently, a third set of 50 CMA-ES runs was performed using NNFF_3_. These runs again found variations of the known overlayer configurations but also added Fig. 5(h) as the overall second lowest energy minimum to the results. The structure is similar to the *pm* eight–ten overlayer structure found by Li *et al.*^12^ It does, however, not fully reproduce the earlier structure where the bridging Ti tetrahedra of the eight ring shares an edge with the substrate whereas the same tetrahedra in (h) shares a corner.

That neither the (h) structure nor the structure proposed by Li were found in the NNFF_1_ CMA-ES run raises the question whether this is due to these structures not being identified as energy minima using the NNFF_1_ or simply a result of the increasing dimensionality of search space with cell size and the stochastic nature of the CMA-ES. To answer this question, we performed a local relaxation with NNFF_1_ starting from the non-optimized (h) structure originally acquired using NNFF_2_ and a reconstructed version of the lowest energy structure proposed by Li *et al.*^12^ We verified that NNFF_1_ correctly identifies the corresponding energy basins on the surrogate PES. From this follows that out of a sufficiently large set of randomly initialized NNFF_1_-backed CMA-ES runs, some would arrive at the structures, without the need for data augmentation. These structures being missed in the original 50 CMA-ES runs we interpret as a result of the dimensionality of the PES being very large for the 5×1 reconstruction. This is also evident in the results obtained for the 3×1 CMA-ES run, where 49 out of 50 runs result in the (a)–(c) structures, as compared to the 4×1 CMA-ES run, where only 12 out of 50 runs result in the (d)-(f) structures. This procedure also verifies that the structure proposed by Li *et al.*^12^ is indeed the lowest energy structure, with a GPAW surface energy 3.2 meV Å^−2^ lower than that of the (g) structure.

Finally, we also re-optimized the three low-energy 5×1 structures with VASP which also identified (g) as the energy minimum, with (h) and (i) having surface energies only around 1 meV Å^−2^ higher.

**Fig. 8:**
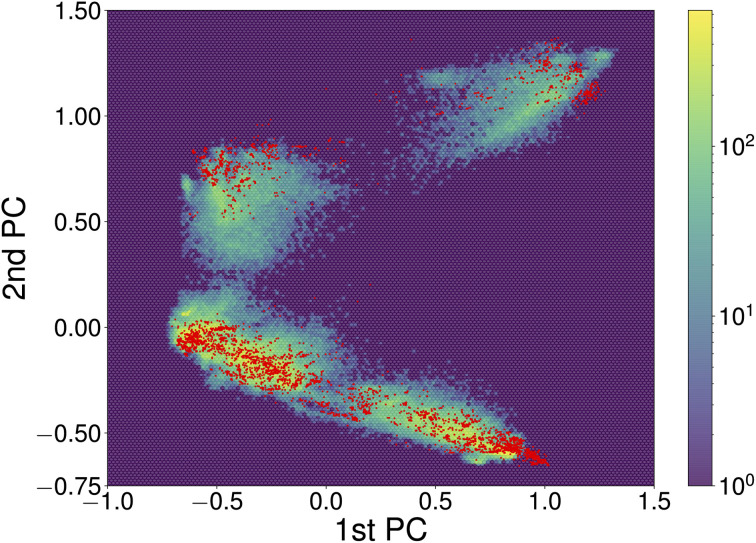
The first two principal components of descriptors of local atomic environments within SrTiO_3_(110) 4×1 training data. The projections of the training data and “Opt” test set are depicted as the hexagonally binned background and red dots, respectively. The principal component analysis (PCA) was performed utilizing the scikit-learn library.^54^ To correct for symmetry and recurring environments, the PCA was limited to local environments on one side of each slab, excluding the environments of atoms within fixed bulk-like layers.

Constructing transferable NNFFs, such that the model can be trained on smaller structures and applied to larger structures or different systems and system sizes is a prominent topic^50,51^ and also part of the original vision behind introducing atomic-descriptor-based NNFFs.^52^ Although a conventional narrative has developed that the performance of ML models is good when they are interpolating between points of the training set and poor when they are used to extrapolate, that informal line of reasoning has not survived rigorous examination. It has been shown that in the high-dimensional spaces commonly explored by ML the probability of a new point falling in the convex hull of the training set is negligible and extrapolation happens constantly.^53^ Whether those new points will be correctly described is therefore more closely related to the functional form and flexibility of the model and the diversity of the data used for training. The prediction of the NNFF can still be expected to perform best for structures with a degree of similarity to the training structures. In our specific case, the good performance of the original 4×1 NNFF, NNFF_1_, can thus be attributed to the good coverage of the space of atomic descriptors afforded by the training structures. To illustrate this, Fig. 8 shows the first two principal components of the descriptors for the 4×1 training data and the same projections of the descriptors for the “Opt” 5×1 test set. The “Opt” data points do fall within or close to the area covered by the local atomic environments represented in the 4×1 training data.

## 4 Summary and conclusions

We successfully combine the covariance matrix adaptation evolution strategy (CMA-ES) and a fully automatically differentiable, high-dimensional neural-network force field (NNFF) to explore the phase diagram of TiO_*x*_ overlayer structures on SrTiO_3_(110) 3×1, 4×1 and 5×1 surfaces. This allows exploiting the stochastic nature of the CMA-ES to perform sets of evolution runs. Thereby the potential energy surfaces (PES) of the systems of interest can be explored more efficiently and to a greater degree than would be possible with DFT. We have thereby arrived at a diverse collection of local energy minima, both reproducing known structures and proposing new stable candidates.

The transferability of the neural-network force field trained solely on the 4×1 surface unit cell structures is demonstrated by comparing to results obtained with neural networks trained on structures from the 5×1 surface unit cell. We attribute the transferability to the diversity of structures generated by the CMA-ES search procedure.

The presented method proves to be capable of thoroughly and efficiently exploring configuration space while greatly reducing the needed computation time. Our formulation of the CMA-ES in a machine-learning context naturally suggests possible extensions to even more complex energy landscapes. Machine-learning models can be used to relax the assumption of Gaussian distribution of candidates and to include intrinsic metrics of the quality of the surrogate model.

## Code availability

A compatible version of NeuralIL, including example scripts for training and evaluation are available.^55^

## Data availability

A dataset containing the CMA-ES founder structures, the overlayers shown in Fig. 5, the trained models and all associated training, validation and test data, is available on Zenodo.^32^

## Conflicts of interest

There are no conflicts to declare.

## Supplementary Material

DD-001-D2DD00072E-s001
