# Supplementary material for: Neural-network-backed evolutionary search for SrTiO3(110) surface reconstructions
Source: Digit Discov. 2022 Aug 26;1(5):703–10. doi: 10.1039/d2dd00072e (PMC9549766; doi:10.1039/d2dd00072e)
Supplement: DD-001-D2DD00072E-s001 [file DD-001-D2DD00072E-s001.pdf]

Electronic Supplementary Material (ESI) for Digital Discovery.
This journal is © The Royal Society of Chemistry 2022

Supplementary Information
Neural-Network-backed evolutionary search for $SrTiO_3(110)$ surface reconstructions

Ralf Wanzenböck,[a] Marco Arrigoni,[a] Sebastian Bichelmaier,[a] Florian Buchner,[a] Jesús Carrete,[a] and Georg K. H. Madsen*[a]

Supplementary figures

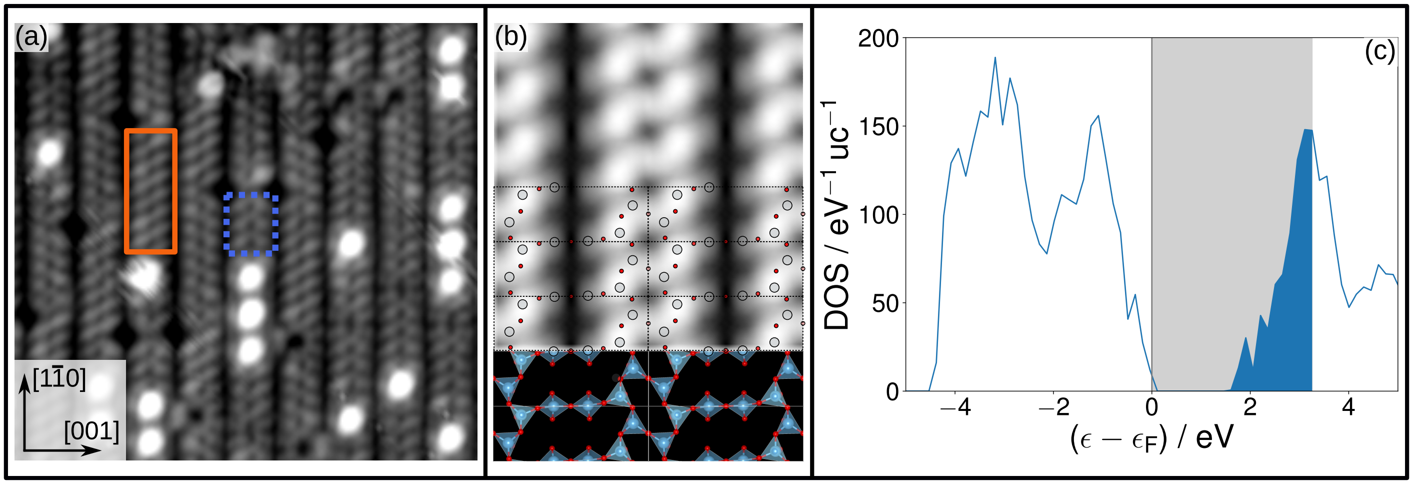

Fig. 1 Comparison of recorded and simulated STM images of the $SrTiO_3(110)$ 4×1 surface. (a) Section of an experimental result courtesy of Zhiming Wang, published in full and annotated in reference[1], shown with added highlights indicating regions with p2 symmetry (solid orange) and pm symmetry (dotted blue). (b) Simulated STM image (Tersoff-Hamann approximation[2] integrating the local density of states up to 3.3 eV above the Fermi level ε_F) of structure 4×1(d) in Fig. 5 of the manuscript, also exhibiting p2 symmetry. (c) Electronic density of states calculated using VASP[3] for the same structure.

[a] *Institute of Materials Chemistry, TU Wien, 1060 Vienna, Austria. E-mail: georg.madsen@tuwien.ac.at*

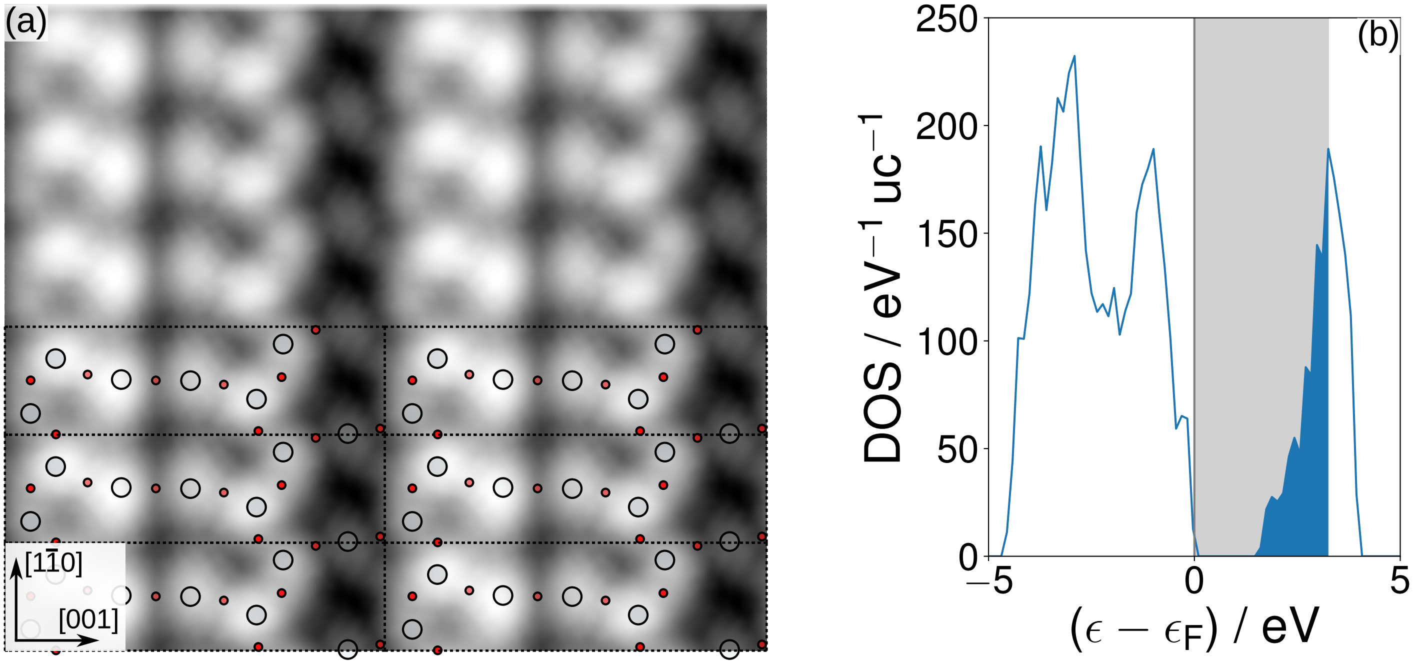

Fig. 2 (a) Simulated STM image (Tersoff-Hamann approximation[2] integrating the local density of states up to 3.3 eV above the Fermi level ε_F) of structure 5×1(i) in Fig. 5 of the manuscript. (b) Electronic density of states calculated using VASP[3] for the same structure.

References

1 Z. Wang, H. Xianfeng, S. Gerhold, M. Schmid, C. Franchini and U. Diebold, *Phys. Rev. B*, 2014, **90**, 035436.

2 J. Tersoff and D. R. Hamann, *Phys. Rev. Lett.*, 1983, **50**, 1998–2001.

3 G. Kresse and J. Furthmüller, *Phys. Rev. B*, 1996, **54**, 11169–11186.
